# The basic amino acids in the coiled-coil domain of CIN85 regulate its interaction with c-Cbl and phosphatidic acid during epidermal growth factor receptor (EGFR) endocytosis

**DOI:** 10.1186/1471-2091-15-13

**Published:** 2014-07-08

**Authors:** Xiudan Zheng, Jing Zhang, Kan Liao

**Affiliations:** 1 From State Key Laboratory of Cell Biology, Institute of Biochemistry and Cell Biology, Shanghai Institutes for Biological Sciences, Chinese Academy of Sciences, Shanghai 200031, China; 2 Current address: Department of Hematology/Oncology, Johann Wolfgang Goethe University, R301 Haus 25 Theodor-Stern-Kai 7, 60590 Frankfurt am Main, Germany

**Keywords:** CIN85, EGFR endocytosis, Phosphatidic acid, Coiled-coil, ESCRT

## Abstract

**Background:**

During EGFR internalization CIN85 bridges EGFR-Cbl complex, endocytic machinery and fusible membrane through the interactions of CIN85 with c-Cbl, endophilins and phosphatidic acid. These protein-protein and protein-lipid interactions are mediated or regulated by the positively charged C-terminal coiled-coil domain of CIN85. However, the details of CIN85-lipid interaction remain unknown. The present study suggested a possible electric interaction between the negative charge of phosphatidic acid and the positive charge of basic amino acids in coiled-coil domain.

**Results:**

Mutations of the basic amino acids in the coiled-coil domain, especially K645, K646, R648 and R650, into neutral amino acid alanine completely blocked the interaction of CIN85 with c-Cbl or phosphatidic acid. However, they did not affect CIN85-endophilin interaction. In addition, CIN85 was found to associate with the internalized EGFR endosomes. It interacted with several ESCRT (Endosomal Sorting Complex Required for Transport) component proteins for ESCRT assembly on endosomal membrane. Mutations in the coiled-coil domain (deletion of the coiled-coil domain or point mutations of the basic amino acids) dissociated CIN85 from endosomes. These mutants bound the ESCRT components in cytoplasm to prevent them from assembly on endosomal membrane and inhibited EGFR sorting for degradation.

**Conclusions:**

As an adaptor protein, CIN85 interacts with variety of partners through several domains. The positive charges of basic amino acids in the coiled-coil domain are not only involved in the interaction with phosphatidic acid, but also regulate the interaction of CIN85 with c-Cbl. CIN85 also interacts with ESCRT components for protein sorting in endosomes. These CIN85-protein and CIN85-lipid interactions enable CIN85 to link EGFR-Cbl endocytic complex with fusible membrane during EGFR endocytosis and subsequently to facilitate ESCRT formation on endosomal membrane for EGFR sorting and degradation.

## Background

CIN85 is identified as a Cbl-interacting protein of 85 kDa and belongs to adaptor/scaffold proteins [1,2]. It consists of three Src homology (SH3) domains, a proline-rich region and a putative α-helical coiled-coil domain at the extreme C-terminal end [1]. As an adaptor protein, CIN85 is involved in Cbl-dependent EGFR internalization, intracellular receptor trafficking, sorting and degradation [3-8]. However, the detailed mechanisms by which CIN85 mediates EGFR endocytic process are not fully defined.

The activation of EGFR by EGF leads to the binding and ubiquitylation of the receptor by c-Cbl, which then recruits CIN85 to EGFR-Cbl complex to initiate the receptor endocytosis [3,9-11]. CIN85, on the other hand, constitutively binds to endophilins, the regulatory proteins that control endocytosis through clathrin-coated pits [3,4,12,13]. Thus, CIN85 tethers EGFR-Cbl complex to the endocytic machinery in an EGF-dependent manner [1]. After internalization, c-Cbl further catalyzes the receptor multi-ubiquitylation and CIN85 mono-ubiquitylation in endosomes [6,14]. The ubiquitylated EGFR is then sorted into multivesicular bodies and fused into lysosomes for degradation [15-17].

EGFR internalization and degradation is enhanced by overexpression of either phospholipase D1 or D2, and inhibited by expression of inactive phospholipase D mutants or by treatment of cells with primary alcohols [18]. The function of phospholipase D in facilitating membrane endocytosis has largely been attributed to its ability to generate phosphatidic acid, a fusogenic lipid that lowers the activation energy required for inward curving of membrane [19,20]. Upon EGF stimulation, phospholipase D is activated in plasma membrane and the production of phosphatidic acid, which enhances membrane fusibility for endocytosis, is increased [19,21].

In our previous study, it is identified that CIN85 binds to phosphatidic acid [22]. This ability enables CIN85 to target phosphatidic acid-enriched membrane. By interacting with EGFR-Cbl complex, endophilins and phosphatidic acid, CIN85 acts as a mediator to bring the receptor, endocytic machinery and fusible membrane together. It is further identified that deletion of the coiled-coil domain blocks CIN85-phosphatidic acid interaction, weakens CIN85-Cbl interaction, dissociates CIN85 from membrane and reduces EGFR degradation [22].

The coiled-coil domain is well-known for its ability to form oligomers in coiled-coil domain-containing proteins, including CIN85 [22-24]. By adding chemical cross-linking reagents, CIN85 is found to form oligomers through its coiled-coil domain [22,23]. Of 70 amino acid residues in the coiled-coil domain, 16 residues are basic amino acids (K or R) [2]. The basic amino acid frequency (23%) is much higher than that in an average protein. Because phosphatidic acid is a negatively charged lipid, the CIN85-phosphatidic acid interaction could be mediated by electric interaction between the negative charge of the lipid and the positive charge of basic amino acids in coiled-coil domain.

In the present study, we found that the basic amino acids in the coiled-coil domain were essential for CIN85-phosphatidic acid interaction. Of 16 K and R residues in the coiled-coil domain, K645, K646, R648 and R650 were the most important. The alanine mutation of these 4 basic amino acids inhibited CIN85-phosphatidic acid interaction, impaired the recruitment of CIN85 by c-Cbl, dissociated CIN85 from EGFR endosomes and reduced EGFR degradation.

## Results

### K645, K646, R648 and R650 in coiled-coil domain are essential for CIN85-phosphatidic acid interaction and membrane association

**Figure 1:**
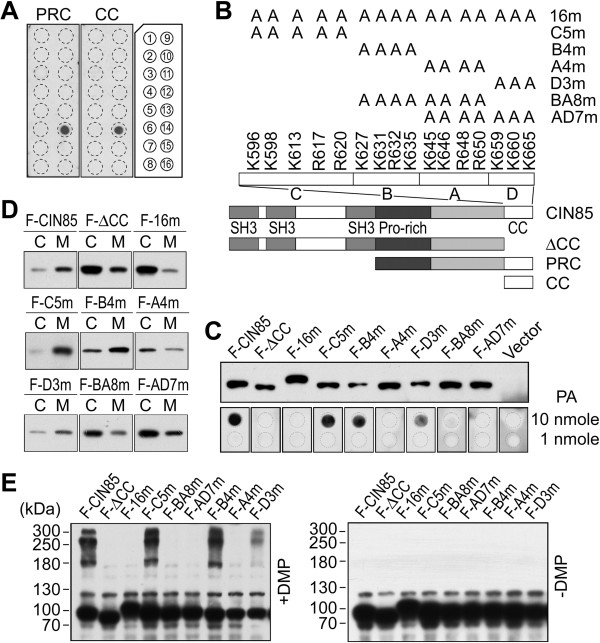
**K645, K646, R648 and R650 in coiled-coil domain are essential for CIN85-phosphatidic acid interaction and membrane association. (A)** Interaction of the coiled-coil domain with phosphatidic acid. The coiled-coil domain (CC) and the C-terminal fragment (PRC) were eGFP-tagged, expressed and purified from HEK293 cells to blot PIP Strip^TM^ membrane. The phospholipids on the PIP Strip^TM^ membrane are: 1, lysophosphatidic acid; 2, lysophosphatidylcholine; 3, phosphatidylinositol; 4, PtdIns(3)P; 5, PtdIns(4)P; 6, PtdIns(5)P; 7, phosphatidylethanolamine; 8, phosphatidylcholine; 9, sphingosine 1-phosphate; 10, PtdIns(3,4)P; 11, PtdIns(3,5)P; 12, PtdIns(4,5)P; 13, PtdIns(3,4,5)P; 14, phosphatidic acid; 15, phosphatidylserine; 16, blank. **(B)** The point mutations of the basic amino acids in the coiled-coil domain and other truncation mutations. The 16 basic amino acids in coiled-coil domain were divided into 4 groups (C5, B4, A4 and D3) and were mutated to alanine (*A*). **(C)** Interaction of CIN85 mutants with phosphatidic acid. Phosphatidic acid (*PA*) was dotted on nitrocellulose membrane at 1 and 10 nmole. The Western blot showed the relative protein level of each mutant used to blot the phosphatidic acid membrane. The mutants were Flag-tagged. **(D)** The association of CIN85 mutants with intracellular membrane vesicles. *M*, the membrane fraction of ultracentrifugation; *C*, the cytosolic fraction of ultracentrifugation. **(E)** Chemical cross-link of CIN85 mutants by dimethylpimelimidate (*DMP*). *+DMP*, cell lysate was treated with DMP; −*DMP*, control cell lysate. The numbers indicate the molecular weight markers.

It was previously identified that the interaction of CIN85 with phosphatidic acid required the C-terminal coiled-coil domain [22]. In fact, coiled-coil domain alone was sufficient to bind phosphatidic acid (Figure 1A). In total 70 amino acid residues of coiled-coil domain there are 11 lysine and 5 arginine residues (Figure 1B). The enriched basic amino acid composition enables the coiled-coil domain to be positively charged in a neutral pH environment and interact with negatively charged proteins or lipids.These 16 basic amino acids naturally form 5 clusters, which were divided into 4 groups for generating mutations: Group C includes K596, K598, K613, R617 and R620; Group B K627, K631, R632 and K635; Group A K645, K646, R648 and R650; Group D K659, K660 and K665 (Figure 1B). They were mutated into alanine, a neutral amino acid, to verify which of these basic amino acids are involved in CIN85-phosphatidic acid interaction (Figure 1B). The mutation of all 16 basic amino acids (CIN85-16m) completely blocked CIN85-phosphatidic acid interaction (Figure 1C). It also inhibited CIN85 oligomerization or the association of CIN85 with membrane vesicles (Figure 1D and E). Essentially, mutation of these 16 basic amino acids exhibited the same effect as deletion of the coiled-coil domain (Figure 1).Mutations of the basic amino acids in individual groups revealed that the basic amino acids in Group A (K645, K646, R648 and R650) were the most important for coiled-coil domain (Figure 1C, D and E). Mutation of these 4 basic amino acids (CIN85-A4m) exhibited the same inhibitory effect as mutation of all 16 basic amino acids. Mutations in other groups (CIN85-C5m, B4m and D3m) did not affect or only partially inhibited the interaction of CIN85 with phosphatidic acid (Figure 1C).

**Figure 2:**
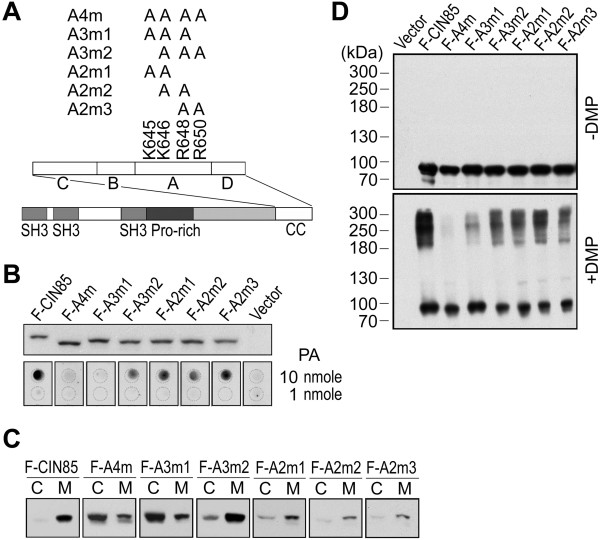
**The net positive charges in group A (K645, K646, R648 and R650) are more important than their positions. (A)** The construction of point mutations in group A. **(B)** Interaction of the mutants with phosphatidic acid. **(C)** The association of the mutants with intracellular membrane vesicles. (**D**) Chemical cross-link of CIN85 mutants by dimethylpimelimidate (*DMP*). The labels in this figure are the same as in Figure 1.

The association of CIN85 with membrane vesicles was most likely mediated by its interaction with phosphatidic acid (Figure 1C and D). Mutations that inhibited CIN85-phosphatidic acid interaction also disrupted CIN85-membrane vesicle association (Figure 1C and D). In fact, CIN85 only associates with liposome containing phosphatidic acid (10%) and phosphatidyl choline (90%), but not liposome of phosphatidyl choline alone [22]. The association with membrane might also facilitate the alignment of CIN85 molecules on membrane surface, enabling them to oligomerize (Figure 1E).Further dissection of the basic amino acids in Group A revealed that the presence of positive charges in this group was more important than the position of these positive charges. Mutations of 2 basic amino acids (K645/646A), (K646/R648A) or (R648/650A) did not affect phosphatidic acid binding, membrane association or oligomerization (Figure 2). Mutations of 3 basic amino acids had mixed effect on the coiled-coil domain. Mutation of K646, R648 and R650 did not affect phosphatidic acid binding, membrane association or oligomerization, whereas mutation of K645, K646 and R648 inhibited these functions (Figure 2B-D). In addition, mutation of either one of these four basic amino acids exhibited no inhibitory effect (results not shown). Overall, there was no single basic amino acid in K645, K646, R648 and R650 that was more prominent than the others.

### K645, K646, R648 and R650 are required for the recruitment of CIN85 by EGFR endocytic complex

**Figure 3:**
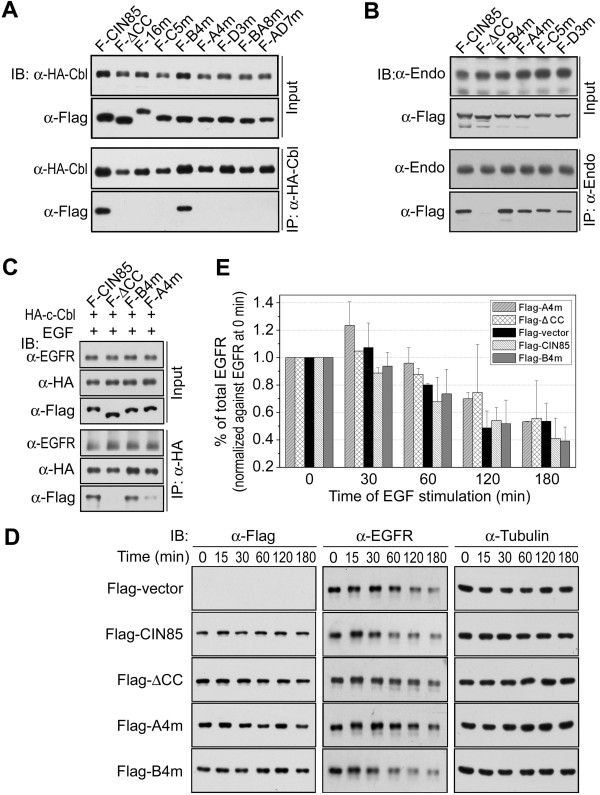
**The mutation of K645, K646, R648 and R650 disrupts CIN85-Cbl interaction and inhibits EGFR degradation. (A)** Interaction of CIN85 mutants with c-Cbl. HA-tagged c-Cbl and Flag-tagged CIN85 mutants were co-expressed in HEK293 cells and HA-Cbl was immunoprecipitated by anti-HA antibody (*IP: α-HA-Cbl*). CIN85 mutants were detected by immunoblot with anti-Flag antibody (*IB*). **(B)** Interaction of CIN85 mutants with endophilins. CIN85 mutants were expressed in COS7 cells and endogenous endophilins were immunoprecipitated by anti-endophilin antibody. **(C)** Interaction of EGFR and c-Cbl in the presence of CIN85 mutants. COS7 cells co-expressing CIN85 mutant and HA-Cbl were serum starved and stimulated with 25 ng/ml EGF for 30 minutes. HA-Cbl was immunoprecipitated by anti-HA antibody. CIN85 mutants and EGFR were detected by immunoblot with anti-Flag antibody and anti-EGFR antibody respectively. **(D)** Inhibition of EGFR downregulation by CIN85-ΔCC or CIN85-A4m. COS7 cells transfected with CIN85, CIN85-ΔCC, CIN85-A4m, CIN85-B4m or blank vector were serum starved and stimulated with 25 ng/ml EGF for indicated times (*0, 15, 30, 60, 120, 180 min*). Cells were harvested and EGFR was detected by Western blot. Tubulin was used as loading control. **(E)** Image gray statistical plot of EGFR downregulation in Figure 3D. Each gray level of EGFR electrophoretic band was normalized against relative EGFR level at 0 min. The final data were averaged from two independent western blot assays.

EGFR endocytosis requires the coordinated interaction among the receptor complex, endocytic machinery and membrane. As an adaptor protein, CIN85 is recruited by c-Cbl to EGFR-Cbl complex [3]. The interaction of CIN85 with c-Cbl is mediated by SH3 domains in CIN85 [22,23]. No direct interaction between c-Cbl and the coiled-coil domain of CIN85 has been identified [22]. Although the coiled-coil domain did not interact with c-Cbl, deletion of this domain led to the impairment of CIN85-Cbl interaction (Figure 3A).Because the basic amino acids in coiled-coil domain were involved in CIN85-lipid interaction (Figure 1), their role in CIN85-Cbl interaction was investigated. As shown in Figure 3A, mutations of the basic amino acids had strong inhibitory effect on CIN85-Cbl interaction. Except mutation in Group B (CIN85-B4m), which did not affect CIN85-Cbl interaction, all the other mutations (CIN85-16m, A4m, C5m and D3m) inhibited CIN85-Cbl interaction (Figure 3A). For most CIN85 mutants, the effect on CIN85-Cbl interaction was parallel to that on CIN85-phosphatidic acid interaction. CIN85-16m, A4m and D3m affected both phosphatidic and c-Cbl binding, while CIN85-B4m inhibited neither of them (Figure 1C and 3A). CIN85-C5m was the only exception that it inhibited the binding to c-Cbl, but not to phosphatidic acid (Figures 1C and 3A).

Through the constitutive CIN85-endophilin interaction, CIN85 links EGFR-Cbl complex with endocytic machinery of clathrin-coated pits [3,13]. Because CIN85-endophilin interaction is mediated by the C-terminal part of CIN85, deletion of coiled-coil domain might disrupt the endophilin interacting region in CIN85 C-terminus [3]. That was the case as deletion of the coiled-coil domain disrupted CIN85-endophilin interaction (Figure 3B). The less disruptive point mutations of the basic amino acids in coiled-coil domain revealed that CIN85-endophilin interaction was independent from the positive charge in coiled-coil domain. There was no difference for endophilin to interact with CIN85, CIN85-A4m, CIN85-B4m or other mutants (Figure 3B).Since CIN85 is recruited by c-Cbl in EGFR-Cbl complex, the interaction between CIN85 and c-Cbl should not affect EGFR-Cbl complex. As shown in Figure 3C, no matter whether the mutants could interact with c-Cbl (CIN85-B4m) or not (CIN85-ΔCC or CIN85-A4m), the presence of CIN85 mutants had no effect on EGFR-Cbl interaction.

### Mutation of K645, K646, R648 and R650 dissociates CIN85 from EGFR endosomal membrane and affects the protein sorting process

**Figure 4:**
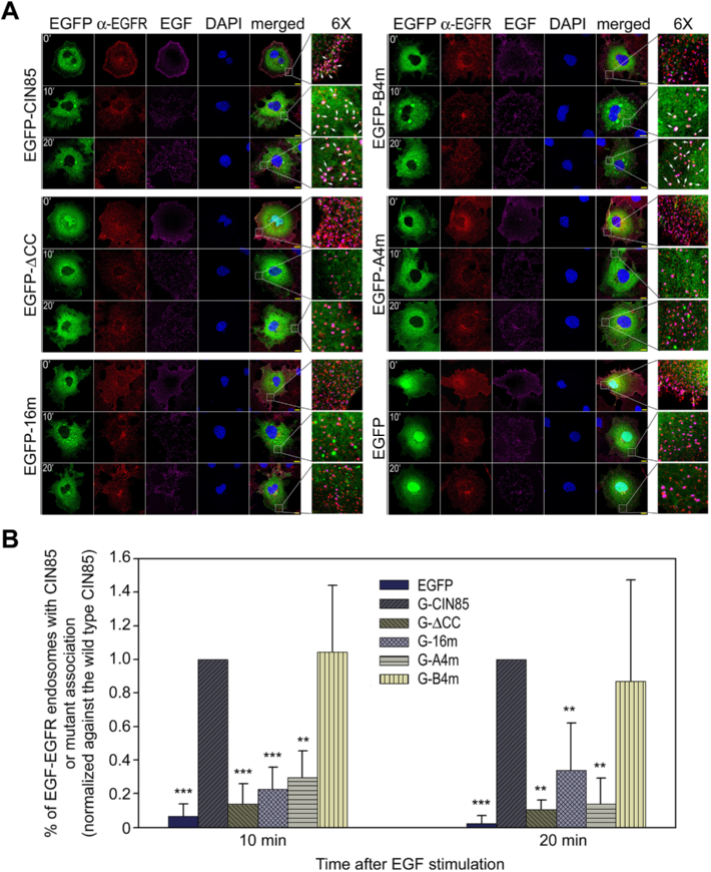
**The mutation of K645, K646, R648 and R650 dissociates CIN85 from EGFR endosome. (A)** EGF endocytosis analysis in eGFP/eGFP-CIN85 mutants transfected COS7 cells. COS7 cells expressing eGFP-tagged CIN85 mutant were incubated with 200 ng/ml Alexa Fluor 647 labeled EGF for 30 min on ice and then shifted to 37°C. At indicated time the cells were fixed and immunostained with anti-EGFR antibody. EGFP-tagged CIN85 mutant was visualized by eGFP fluorescence and the labeled EGF was visualized by the fluorescence of Alexa Fluor 647. A selected area is enlarged 6 times to reveal the details. The arrows point the white dots which are the colocalization of EGF (purple), EGFR (red) and eGFP-CIN85 mutant (green). The bar is 10 μm. **(B)** Plot of the percentage of EGF-EGFR endosomes with eGFP-CIN85 or mutant association. For each mutant, 10 cells were used to calculate white dots and total EGF-EGFR endosomes, and the percentage was normalized against the average percentage in eGFP-CIN85 expressing cell. Student’s *t*-test was performed. The *P* values between eGFP-CIN85 expressing cells and eGFP expressing cells or eGFP-CIN85 mutants expressing cells were ***9.34E-05 (10 min) and ***0.000272 (20 min) for eGFP; ***0.000187 (10 min) and **0.001979 (20 min) for eGFP-ΔCC; ***0.000252 (10 min) and **0.007448 (20 min) for eGFP-16 m; **0.001818 (10 min) and **0.002121 (20 min) for eGFP-A4m; 0.329283 (10 min) and 0.739651 (20 min) for eGFP-B4m.

In COS7 cells expressing CIN85 null-function mutant (CIN85-ΔCC or CIN85-A4m), EGF-stimulated EGFR degradation was decreased, whereas in cells expressing neutral mutant (CIN85-B4m) or wild type CIN85 the ligand-stimulated receptor degradation was not affected (Figure 3D and E). However, no difference in EGFR internalization was observed in COS7 cells expressing eGFP, CIN85 or CIN85 mutant (neutral or null-function) (Figure 4A). Thus, the expression of CIN85 null-function mutant did not inhibit the receptor internalization *per se* (Figure 4A). It only reduced the degradation of the internalized EGFR (Figure 3D and E). The inhibition of EGFR degradation but not EGFR internalization suggested that these CIN85 null-function mutants interfere with EGFR sorting/degradation process.

After EGFR internalization, CIN85 or the neutral mutant CIN85-B4m was found to be associated with EGFR endosomes (Figure 4A). Both of them exhibited similar endosome binding (Figure 4B). In contrast, CIN85 null-function mutants (CIN85-ΔCC, CIN85-16m or CIN85-A4m) did not associated with EGFR endosomes (Figure 4A and B). The internalized EGFR is sorted by the assembly of ESCRT (Endosomal Sorting Complex Required for Transport) on endosomal membrane [25]. Vps (vacuolar protein sorting) is a group of ESCRT components that are recruited to endosomal membrane by adaptor proteins [26]. It is possible that CIN85 on endosomal membrane interacts with these ESCRT components to facilitate ESCRT assembly.

**Figure 5:**
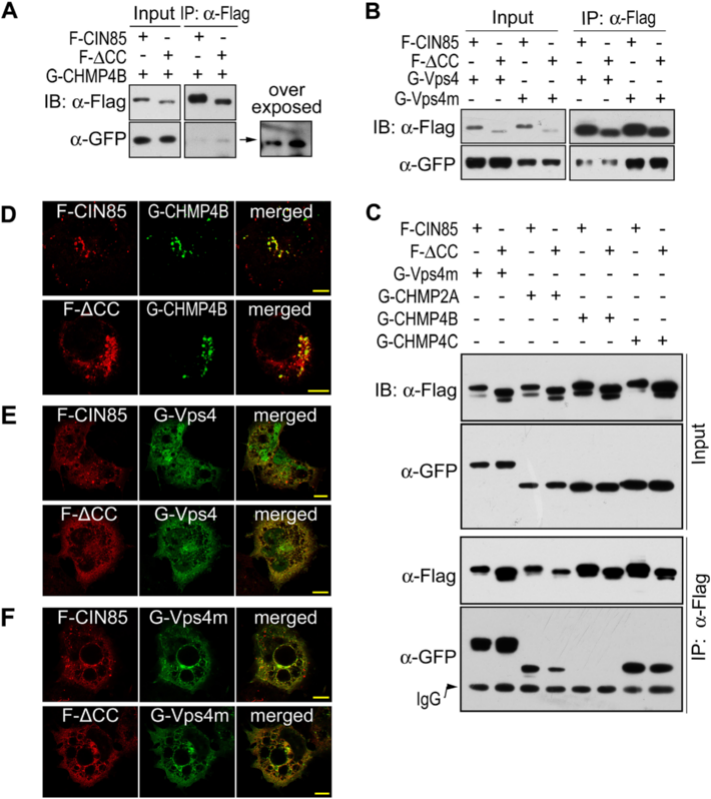
**Interactions of CIN85 with Vps proteins. (A)** Interactions of CIN85 and CIN85-ΔCC with CHMP4B. Flag-tagged CIN85 and eGFP-tagged CHMP4B were co-expressed in HEK293 cells and Flag-CIN85 was immunoprecipitated by anti-Flag antibody. CHMP4B in immunoprecipitated sample was detected by Western blot. **(B)** Interactions of CIN85 and CIN85-ΔCC with Vps4 and Vps4m (ATPase negative mutant). Flag-tagged CIN85 and eGFP-tagged Vps4 were co-expressed in HEK293 cells and Flag-CIN85 was immunoprecipitated by anti-Flag antibody. Vps4 in immunoprecipitated sample was detected by Western blot. **(C)** Interactions of CIN85 and CIN85-ΔCC with different Vps proteins. The tagged CIN85 and Vps protein were co-expressed in HEK293 cells and CIN85 was immunoprecipitated. Vps proteins were detected by Western blot. **(D)** Co-localization of CIN85 and CIN85-ΔCC with CHMP4B. Flag-tagged CIN85 was co-expressed with eGFP-tagged CHMP4B in HEK293 cells. Cells were fixed with 4% PFA and observed by confocal microscopy. Τhe bar is 10 μm. **(E, F)** Co-localization of Flag-tagged CIN85 and CIN85-ΔCC with eGFP-tagged Vps4 and Vps4m.

**Figure 6:**
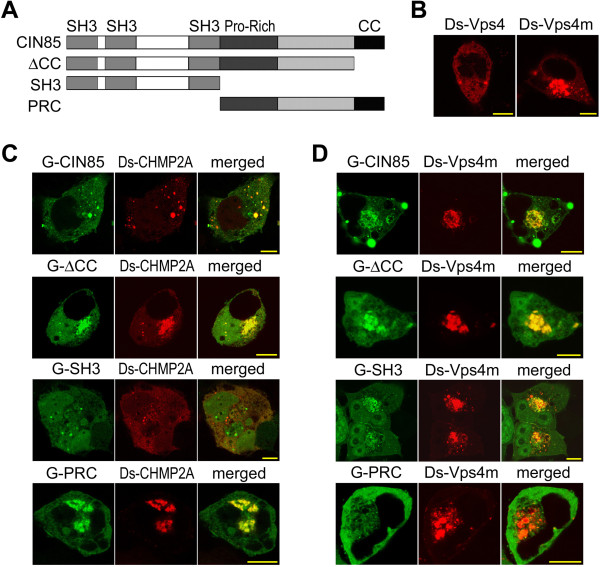
**Cellular colocalization of Vps4 and CHMP2A with CIN85 domains.** Τhe bar is 10 μm. **(A)** Construction of fusion proteins of CIN85 fragment. CIN85 fragment was tagged with eGFP. **(B)** Intracellular localization of Vps4 and Vps4m (ATPase negative mutant). The proteins were tagged with DsRed and expressed in HEK293 cells. **(C)** Colocalization of CIN85 fragment with CHMP2A. EGFP-tagged CIN85 mutant and DsRed-tagged CHMP2A were co-expressed in HEK293 cells. The proteins were visualized by the tagged fluorescence protein. **(D)** Colocalization of CIN85 fragment with Vps4m.

In our previous study, CIN85 was found to be co-localized with Vps4, Vps4m and CHMP4B (Vps32B) [22]. Moreover, coiled-coil domain deletion (CIN85-ΔCC) did not affect their cellular co-localization (Figure 5D-F). There were also protein interactions between CIN85 and Vps4, Vps4m and CHMP4B (Vps32B) (Figure 5A and B). Vps4 is a cytoplasmic ATPase that is recruited to ESCRT-III to regulate ESCRT dissociation [27]. It weakly interacted with CIN85 (Figure 5B) [22]. However, its ATPase-negative mutant, Vps4m that constitutively associates with endosomal membrane [28], dramatically increased its interaction with CIN85 (Figure 5B). For both Vps4 and Vps4m, their interaction with CIN85 or cellular co-localization was independent from the coiled-coil domain (Figure 5B,C,E and F). In addition, Vps4m appeared to be associated with intracellular membrane vesicles, whereas the wild type Vps4 was mostly cytoplasmic protein (Figure 6B).

There was only weak interaction between CIN85 and CHMP4B (Figure 5A and C), although they were co-localized in cells (Figure 5D) [22]. In comparison to the interactions with other ESCRT proteins [Vps4m, CHMP2A (Vps2A) and CHMP4C (Vps32C)], CIN85-CHMP4B interaction was negligible (Figure 5C). Little amount CHMP4B was co-precipitated with either CIN85 or CIN85-ΔCC (Figure 5A). On the other hand, CHMP2A (Vps2A) and CHMP4C (Vps32C), two core members in ESCRT-III that are assembled in sequential manner on endosomal membrane, interacted with CIN85 strongly (Figure 5C).

In ESCRT complex not all the components are in similar position to contact with CIN85 [25]. The domains of CIN85 involved in the interaction varied with different Vps proteins. The interaction of CIN85 with CHMP2A was mediated by the C-terminus containing the proline-rich region, as CHMP2A was colocalized with CIN85 C-terminus, but not N-terminal SH3 domains (Figure 6C). In contrast, the interaction between CIN85 and Vps4 was most likely mediated by the N-terminal SH3 domains (Figure 6D). Vps4m was colocalized with N-terminal SH3 domains, but not C-terminus containing the proline-rich region (Figure 6D). Whatever the differences were in the interactions between CIN85 and Vps proteins, they were all independent from the coiled-coil domain (Figure 5C). As CIN85 with mutations in coiled-coil domain (CIN85-ΔCC, CIN85-16m or CIN85-A4m) dissociated from EGFR endosomes, their interaction with Vps proteins in cytoplasm prevented the Vps proteins to interact with EGFR-Cbl-CIN85 complex on endosomal membrane. Thus, the presence of exogenous CIN85 mutants interfered with EGFR sorting process in endosomes and less EGFR was sorted to lysosome for degradation.

## Discussion

The recruitment of CIN85 by c-Cbl in EGFR-Cbl complex has been indicated as the initial step for EGFR internalization [3]. The interaction of CIN85 with c-Cbl is mediated by the SH3 domains in CIN85 and a novel proline-arginine motif present in the distal carboxyl-terminus of Cbl [3,22,23,29]. Interestingly, c-Cbl can bind to wild type CIN85 or CIN85 fragment containing only the SH3 domains (CIN85-SH3), but cannot bind to CIN85 with coiled-coil domain deletion (CIN85-ΔCC) [3,22]. The SH3 domains in CIN85-ΔCC appear to be blocked for c-Cbl interaction, possibly due to the conformational change in CIN85 lacking the coiled-coil domain [30]. However, the detailed mechanism by which the deletion of coiled-coil domain affects CIN85-Cbl interaction is still not clear. It is possible that without coiled-coil domain the proline-rich region in CIN85 may engage in auto-interaction with the SH3 domains to prevent them to bind c-Cbl [31]. The difference of CIN85-SH3 and CIN85-ΔCC in c-Cbl binding leads to different effects on EGFR internalization. CIN85-SH3 has a dominant negative effect on EGF stimulated EGFR internalization [11], whereas CIN85-ΔCC exhibits no inhibitory effect on EGFR internalization (Figure 4). The interaction of CIN85-SH3 with c-Cbl competes off the binding of functional CIN85 to EGFR-Cbl complex. Due to the lack of C-terminal endophilin-interacting domains, CIN85-SH3 cannot tether EGFR-Cbl complex with endocytic machinery, blocking endophilin-mediated EGFR endocytosis. CIN85-ΔCC, on the other hand, cannot interact with c-Cbl and is a bystander to EGFR-Cbl complex. The endogenous CIN85 can still be recruited by c-Cbl to form EGFR-Cbl-CIN85 complex, which then recruits endocytic proteins for receptor internalization. Thus, binding of CIN85 mutant to c-Cbl is essential for the dominant negative inhibition of EGFR internalization.

The point mutations in the coiled-coil domain minimize the disruptive effect of protein truncation [22]. In fact, the point mutation of the basic amino acids in the coiled-coil domain (CIN85-16m) is sufficient to disrupt CIN85-Cbl interaction (Figure 3A). It not only inhibits CIN85-Cbl interaction, but also blocks CIN85-phosphatidic acid interaction (Figures 1 and 3). The results from the mutations of basic amino acids in coiled-coil domain support the electric charge interaction between phosphatidic acid and CIN85 (Figure 1). K645, K646, R648 and R650 out of 16 basic amino acids in coiled-coil domain are the most important in phosphatidic acid binding, membrane association and c-Cbl interaction (Figure 1 and 3). Mutation of these four basic amino acids (CIN85-A4m) is sufficient to disrupt every CIN85 interactions except CIN85-endophilin interaction (Figure 3B).

The interactions with c-Cbl and phosphatidic acid enable CIN85 to couple EGFR endocytic complex with fusible membrane. The results from study of phospholipase D activation and EGFR endocytosis support this kind of association, since EGF stimulation induces the activation of phospholipase D as well as EGFR internalization [18]. Phosphatidic acid is generated by phospholipase D in response to EGF or other growth factors [19-21]. The activation of phospholipase D increases phosphatidic acid concentration in plasma membrane, while the stimulation of EGFR triggers the c-Cbl binding to initiate the endocytic complex formation [3]. By bridging these two events, CIN85 facilitates the endocytic complex formation at membrane area enriched with fusogenic lipid.

Reports have shown that knockdown of CIN85 by RNA interference has no significant effect on EGFR internalization, but rather delays the degradation of internalized EGFR [8,11]. It is further identified that the internalized EGFR is recycled rather than degraded in CIN85-depleted cells [11]. Depletion of CIN85 shifts the trafficking of internalized EGFR from the degradative to the recycling pathway. It seems to be that CIN85 is required for the sorting of the internalized EGFR. And CIN85 independent endocytic pathways are involved in EGFR internalization in CIN85-depleted cells.

**Figure 7:**
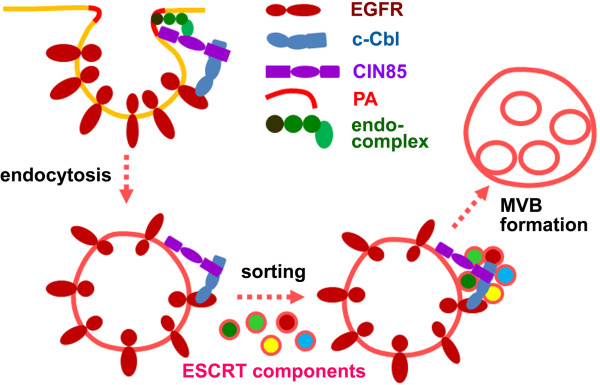
**Illustration of EGFR endocytosis process.** The functions of CIN85 during EGFR internalization and sorting are depicted in this illustration. It is not a comprehensive model for EGFR endocytosis process, rather a model for understanding the functions of CIN85 in EGFR endocytosis process. *PA*, phosphatidic acid; *endo-complex*, endophilins and endocytic protein complex; *MVB*, multi-vesicular bodies.

After EGFR internalization, CIN85, associated to EGFR-Cbl complex, appears on endosomal membrane (Figure 4). Its interaction with ESCRT components will facilitate the assembly of ESCRT on endosomal membrane (Figure 5). CIN85 mutants (CIN85-ΔCC, CIN85-16m and CIN85-A4m) that do not bind to c-Cbl or phosphatidic acid lose their ability to associate with EGFR-positive endosomes (Figure 4). These mutants, however, can still interact with ESCRT components in cytoplasm, equivalent to depleting ESCRT components for assembly on endosomal membrane and resulting in the inhibition of EGFR sorting for degradation (Figure 5). Similar result has been obtained by knocking down ESCRT protein. For example, the depletion of CHMP3 (Vps24, a component of ESCRT-III) in mammalian cells severely inhibits EGFR degradation, but the termination of EGFR membrane signal remains unaffected [32].As an adaptor protein, CIN85 acts as a mediator in EGFR endocytosis and sorting process. Its ability to interact with multiple proteins and fusogenic lipid also makes CIN85 a mediator between proteins and lipid. Membrane receptor endocytosis, sorting and degradation are events of coordinated protein and membrane interactions. CIN85 appears to be an important match-maker in these events (Figure 7).

## Conclusions

During EGFR endocytosis, CIN85 tethers EGFR, phosphatidic acid and clathrin-coated pits to facilitate the receptor internalization. The interaction of CIN85 with EGFR-Cbl complex and phosphatidic acid targeted the internalized proteins to membrane areas enriched with fusogenic lipid. The coiled-coil domain, especially the basic amino acids in the domain, is the key regulatory element for the interactions of CIN85 with EGFR-Cbl as well as phosphatidic acid. After endocytosis, CIN85 in association with EGFR-Cbl complex on endosomal membrane recruits several ESCRT components to endosome for ESCRT assembly. Then the ESCRT complex on endosomal membrane induces the formation of multi-vesicular bodies to sort the internalized receptor for degradation or recycling.

## Methods

### Ethics statement

All results of this research were based on cultured HEK293 or COS7 cells lines. Neither human (human subjects, human material or human data) nor animals (vertebrates or any regulated invertebrates) were used in this experimental research.

### Materials

Anti-EGFR, anti-Cbl and anti-endophilins antibodies were purchased from Santa Cruz. Anti-CIN85 monoclonal antibody was from Upstate. Anti-flag monoclonal antibody, phosphatidic acid, DMP (dimethylpimelimidate) and horse radish peroxidase-conjuated secondary antibodies were from Sigma. EGF, Alexa Fluor 647 labeled EGF, PIP Strips™ membrane and fluorescence labeled secondary antibodies (Alexa Fluor 488/546 conjugates) for immunofluorescence were the products of Life Technologies. The leica laser scanning confocal microsystem, including the leica TCS SP2 confocal microscope, Leica confocal scanner, and Leica confocal acquisition software were used with the HCX PL APO 1bd. BL 63.0 X/1.4 oil objective at 1.4 numerical aperture at a working temperature of 22°C.

### Plasmids construction

The truncation and point mutations of CIN85 were constructed by polymerase chain reaction amplification and the mutants were tagged with Flag-tag or eGFP at the N-terminus of the fusion protein. CIN85-ΔCC was the deletion of amino acid 601 to 665. CIN85-PRC was the fragment of amino acid 334 to 665. CIN85-CC was the coiled-coil domain of amino acid 594 to 665. CIN85-SH3 was the N-terminus of amino acid 1 to 331. The 16 basic amino acids in CIN85 from amino acid 596 to 665 (K596, K598, K613, R617, R620, K627, K631, R632, K635, K645, K646, R648, R650, K659, K660 and K665) were divided into several groups and mutated to alanine residues.

### Protein expression, immunofluorescence staining, immunoprecipitation and western blot

CIN85 mutant plasmid was transiently transfected into HEK293 or COS7 cells and experiments were performed in 48 hours. For immunofluorescence staining, cells were fixed in 4% paraformaldehyde (PFA) in phosphate buffered saline (PBS). EGFP fusion proteins and Fluor-labeled EGF were directly visualized by confocal microscope. Other proteins were stained with appropriate primary antibodies and Fluor-labeled secondary antibodies. For immunoprecipitation, two plasmids in 1:3 ratio (primary immunoprecipitated protein/co-precipitated protein) were co-transfected into cells and the cells were lysed in a buffer containing 1% Triton X-100, 10% glycerol, 20 mM Hepes, pH7.4, 150 mM NaCl, 1 mM EDTA, 1 mM PMSF and 2 μl/ml protease inhibitor cocktails 1 and 2. The immunoprecipitation and western blots were conducted as described previously [33].

### Cell fractionation for intracellular membrane vesicles

Cells were homogenized in isotonic buffer containing 20 mM Hepes, pH 7.4, 0.25 M sucrose, 1 mM EDTA, 2.5 mM dithiothreitol, 1 mM PMSF and 2 μl/ml protease inhibitor cocktails 1 and 2. The homogenized cell lysate was centrifuged at 12,000 × g for 15 min. The pellet was discarded and the supernatant was then ultracentrifuged at 100,000 × g for 90 minutes to separate intracellular membrane vesicles and cytoplasm.

### Phosphatidic acid association

The eGFP-tagged CIN85-PRC or CIN85-CC was expressed in HEK293 cells and purified by immunoprecipitation. The immunopurified fusion protein was incubated with PIP Strips^TM^ following the manufacturer’s instruction and detected by immunoblot with anti-eGFP antibody. The phosphatidic acid membrane was prepared by dotting nitrocellulose membrane with phosphatidic acid in chloroform solution. CIN85 mutants expressed in HEK293 cell were prepared by homogenizing the cell in buffer containing 20 mM Hepes, pH7.4, 150 mM NaCl, 2 mM MgCl_2_, 15 mM KCl, 1 mM EDTA, 1 mM PMSF and 2 μl/ml protease inhibitor cocktails 1 and 2. The mixture was then centrifuged at 12,000 × g for 15 min and the supernatant was used to blot the phosphatidic acid membrane. The proteins were detected by anti-Flag antibody.

### Downregulation and internalization of EGFR

COS7 cells were transfected with CIN85 mutant plasmid, serum-starved and stimulated with 25 ng/ml EGF. The cells were harvested at various time points and EGFR protein was detected by Western blot. The analysis of EGFR internalization followed the protocol developed by Doyotte et al. [34] and Raiborg et al. [35]. Briefly, COS7 cells were transfected with CIN85 mutants. After 72 hours, the cells were serum-starved overnight and incubated with Alexa Fluor-labeled EGF (200 ng/ml in Eagle’s minimal essential medium supplemented with 20 mM Hepes and 2 mg/ml bovine serum albumin) on ice for 30 minutes. Cells were then washed with pre-warmed binding buffer (37°C) three times and incubated in binding buffer for indicated length of time before fixation with 4% PFA.

### Chemical cross-link by dimethylpimelimidate (DMP)

HEK293 cells expressing CIN85 mutants were lysed in 0.2 M triethanolamine (pH 8.0). 100 μM DMP was added to the lysate and the mixture was incubated for 1 hour at 4°C. The cross-linking reaction was terminated by adding 100 mM Tris–HCl (pH 7.5). The samples were subjected to SDS-PAGE for western blot.

## Abbreviations

CIN85: Cbl-interacting protein of 85 kDa; Cbl: Casitas B-lineage lymphoma proto-oncoprotein; EGFR: Epidermal growth factor receptor; PA: Phosphatidic acid; ESCRT: Endosomal sorting complex required for transport; CC: Coiled-coil domain; SH3: Src homology domain; ΔCC: Coiled-coil domain deletion; PRC: Proline-rich C-terminal region; Vps: Vacuolar protein sorting; CHMP: Charged multivesicular body protein.

## Competing interests

The authors declare that they have no competing interests.

## Authors’ contribution

XZ performed plasmid construction, cell transfections, cell fractionation, phosphatidic acid association, chemical cross-link, localization immunofluorescence, immunoprecipitations, downregulation and internalization assay of EGFR, and statistical analysis. JZ performed plasmid construction, cell transfections and localization immunofluorescence. KL conceived of the study, participated in its design and coordination and drafted the manuscript. The final manuscript was read and approved by all authors.
